# User Experience of Older Adults With an Age-Friendly Transportation Planning E-Tool: Scoping Review

**DOI:** 10.2196/63273

**Published:** 2025-07-28

**Authors:** Sara Bahrampoor Givi, Mireille Gagnon-Roy, Hélène Pigot, Véronique Provencher

**Affiliations:** 1 Research Center on Aging, Centre intégré universitaire de santé et de services sociaux de l'Estrie - Centre hospitalier universitaire de Sherbrooke, 1036 Rue Belvédère S, Sherbrooke, QC, J1H 4C4, Canada, 1 8197802220 ext 45657; 2 Faculty of Letters and Human Sciences, Université de Sherbrooke, Sherbrooke, QC, Canada; 3 Faculty of Medicine and Health Sciences, Université de Sherbrooke, Sherbrooke, QC, Canada; 4 Department of Computer Science, Université de Sherbrooke, Sherbrooke, QC, Canada; 5 School of Rehabilitation, Université de Sherbrooke, Sherbrooke, QC, Canada

**Keywords:** older adults, transportation, user experience, E-tool, review, design

## Abstract

**Background:**

Aging is associated with various challenges, especially concerning mobility. Transportation planning e-tools are currently available to provide older adults with real-time travel information and help them choose trip options. However, many older adults find them challenging to use, as they are not tailored to their specific needs, such as lack of accessibility to the different means of transportation. It is necessary to identify knowledge gaps about design based on older adults’ experience using transportation planning e-tools.

**Objective:**

This study aims to identify knowledge gaps regarding the user experience design and its evaluation for older adults using transportation planning e-tools.

**Methods:**

A scoping review of the scientific literature was conducted, based on Arksey and O’Malley’s guidelines. The search covers sources published in English from January 2002 to October 2022 through 7 scientific databases, including MEDLINE, AgeLine, CINAHL, SCOPUS, ProQuest, IEEE Explore, and TRID (Transportation Research International Documentation), and was updated in October 2023. Data selection and extraction were performed by the first author and were co-validated by 2 co-authors. The identified sources were analyzed based on source characteristics (authors, year of publication, title of the article, source of article, country, and context of the studies), the purpose of the studies (objectives and study orientation), and the mapping of the methodology and evaluation of user experience (design approach, setting, type of data collection and analysis, type of usability evaluation, and sample size). Both descriptive-analytical methods and thematic analysis were used to analyze the categorized data.

**Results:**

Overall, 1905 sources were identified through databases, and 40 sources were selected for full-text analysis. Data analysis revealed in recent years that there has been significant growth in e-tools designed for older adults, but only 2 studies were related to the field of transportation. In total, 12 studies aimed to evaluate user experience, and 22 studies focused on a user-centered design approach. Most of these studies (n=31) were carried out in a laboratory setting, using summative usability evaluation (user-based testing). The System Usability Scale (SUS) was the most prevalent tool (15 studies) to measure user efficiency and satisfaction. However, no studies have been found that specifically aim to improve the mobility experience of older adults using an age-friendly transportation planning e-tool in a real-life context.

**Conclusions:**

There is a lack of studies assessing older adults’ experience when using transportation planning e-tools in real-life situations. To bridge this gap, a participatory approach is necessary to better consider the needs of older adults in a real-life context and create age-friendly design guidelines for the development of transportation planning e-tools. This will not only enhance their user experience in trip planning but also promote their social engagement by improving their mobility.

## Introduction

### Background

The population of individuals aged 65 years and above is projected to increase from 900 million in 2015 to 2 billion by 2050 [1]. Recent data reported that 37.8% of Canadians over the age of 65 years live with a disability, which increases mobility issues in their daily travel [2]. Indeed, some age-related changes may impede the use of transportation for many older adults to participate in meaningful activities. Decreased vision [3-5] and hearing loss [6,7] make it difficult to see other road users (especially at night), recognize traffic signs (reading text and distinguishing colors) while driving, and hear high-frequency sounds when crossing the street. In addition, decreased flexibility, range of motion, and grip strength can limit driving control and impair driving safety [7], which makes it difficult for them to get around. Therefore, it is necessary to support the ability of older adults to know and use transportation options that are tailored to their needs so that they continue to be able to leave their homes, which is an essential characteristic of social participation [8].

Transportation-planning e-tools are currently available to provide older adults with information about transportation and trip options. These tools can take the form of websites or applications designed to assist users in planning, personalizing, and managing their travel. However, many older adults find them challenging to use. With increased aging, reduced dexterity and lower vision make it difficult to locate the necessary information on the screen or click on the appropriate button, which can impede their experience when planning travels in the community [9]. Specifically, visual decline also impacts the contrast sensitivity in using travel planning e-tools (eg, recognizing the elements in maps or e-tools). The decline of fine motor skills [4] may interfere with the use of electronic devices that allow movement planning. Moreover, lower executive abilities can lead to difficulty in planning travel and understanding public transportation options [10]. Besides, some older adults experience difficulty in travel planning due to their lack of digital literacy [11], which may raise challenges, such as access to the up-to-date flow of information for travel planning [12]. The interface of the developed tool should thus consider these age-related changes and the variability in numerical literacy [13] in planning trips. When it comes to designing an interface, user experience is essential [14].

User experience is defined as an “iterative set of decisions leading to a successful outcome with an interactive tool, as well as a productive and satisfying process while arriving at this outcome” [15]. According to Hassenzahl and Tractinsky [16], it is created by the interaction of the user’s internal state, the characteristics of the designed system, and context. It should be noted that 2 other concepts should be introduced in relation to user experience: user interface and usability. User interface is “the part of technology that people interact with. The interaction between a computer and a user is a two-way interaction” [17]. Therefore, user interface design refers to “the iterative set of decisions leading to a successful implementation of an interactive tool” [15]. Usability refers to the fact that “when a product or service is truly usable, the user can do what she or he wants to do, the way she or he expects to be able to do it, without hindrance, hesitation or questions” [18]. Hassan and Galal-Edeen [19] argue that usability is a subset of user experience, and none alone can determine the degree of conformity of a product or service to the expectations and needs of its users. The usability criteria include usefulness, efficiency, effectiveness, satisfaction, learnability, accessibility, memorability, and error tolerance. Considering these definitions in the framework of this study, the user experience refers to the interaction of older adults with the aesthetic and functional aspects of the transportation planning e-tool to plan a trip that satisfactorily meets their needs and preferences.

A co-design design is a participatory approach that can be relevant to older adults to address their challenges in planning a trip and improve the user experience when using e-tools. Participatory design refers to “a process of investigating, understanding, reflecting upon, establishing, developing, and supporting mutual learning between multiple participants in collective reflection-in-action” [20]. Understanding the interactions and collaboration of older adults in co-design approaches such as participatory design [20] is an important aspect of user experience design [21] as older adults’ needs and physical condition can affect their participation in the design process. Participatory design could help evaluate user experience. As there is a lack of research on user experience design for older adults [22], it is essential to support older adults’ mobility through travel options that are tailored to their needs and preferences by identifying knowledge gaps regarding the user experience design of older adults when using a transportation planning e-tool.

### Context and Objective

The aim of this scoping review is to identify knowledge gaps regarding the user experience design and its evaluation for older adults using transportation planning e-tools.

## Methods

### Study Design

This scoping review was based on Arksey and O’Malley’s [23] framework with the following stages: (1) identifying the research question; (2) identifying the relevant sources; (3) selecting the studies; (4) charting the data; and (5) collecting, summarizing, and reporting results.

### Identifying the Research Question

The question was formulated using a broad approach to ensure sufficient breadth during the literature search. The question is: What is known about the user experience design, co-design, and its evaluation for older adults using transportation-planning e-tools to move around in the community? This scoping review followed the PCC framework: (P) older adults, (C) user experience design, co-design, and usability evaluation, and (C) transportation-planning e-tools.

### Identifying the Relevant Sources

This stage consisted of searching scientific literature published in English from January 2002 to October 2022, with an updated search conducted on October 3, 2023. The search was performed across 7 databases: MEDLINE (PubMed, NCBI), AgeLine (EBSCOhost), CINAHL Plus with Full Text (EBSCOhost), SCOPUS (Elsevier), ProQuest Dissertations and Theses (ProQuest), IEEE Xplore (IEEE Xplore Digital Library), and TRID (Transportation Research Board).

Databases and keywords were selected and confirmed during a multi-step process. First, the keywords and databases were selected by two of the authors (SBG and VP). Then, an experienced librarian (FL) reviewed and refined them, ensuring that the selected terms and databases were relevant to the research question. As part of the search strategy confirmation, FL provided feedback aligned with the PRESS 2015 Guidelines [24], offering expert guidance on refining keywords, optimizing Boolean operators, and using controlled vocabulary where available. These adjustments helped ensure both the accuracy and comprehensiveness of the search. Some decisions were made to retrieve the largest number of relevant sources. During the pretesting of the research strategy, incorporating transportation-related keywords alongside other search terms significantly reduced the number of relevant studies. To address this issue, the librarian recommended removing these keywords and adding the TRID (Transportation Research International Documentation) and IEEE Xplore databases. This adjustment ensured a more comprehensive search and improved the scope of the review. Finally, the keywords and databases were confirmed by a senior researcher (HP), an experienced professor in the field of user experience. The full search strategies, including controlled vocabulary and keywords, are detailed in Table 1. To ensure comprehensive coverage, we first identified controlled vocabulary terms from each database’s thesaurus (eg, MeSH in PubMed, CINAHL Headings, and IEEE Xplore’s indexing terms). Based on these terms, we then created a structured list of keywords for each concept to capture both indexed and natural variations of relevant terms.

**Table 1.** Concepts and keywords.

Concept	Controlled Vocabulary (by Database)	Keywords
User experience	User-computer interface (MeSH—MEDLINE, PubMed) Usability testing (CINAHL headings) Human-computer interaction (IEEE Thesaurus)	“User Interface” OR “User interface design” OR “UI” OR “UI design” OR “User experience” OR “User experience design” OR “UX” OR “UX design” OR “Usability”
Aging	Aged (MeSH—MEDLINE, PubMed) Older adults (CINAHL headings) Older adults (TRB thesaurus)	“Older adult” OR “Old people” OR “Elderly” OR “Senior” OR “Aging” OR “Normal aging” OR “Frailty”
E-tool	Mobile applications (MeSH—MEDLINE, PubMed) Software applications (IEEE thesaurus) Web applications (CINAHL headings)	“Website” OR “Application” OR “E-tool” OR “Web application” OR “App” OR “Web”
Co-design	User-centered design (MeSH—MEDLINE, PubMed, CINAHL, SCOPUS) Participatory design (IEEE thesaurus)	“Co-design” OR “Participatory Design” OR “Co-operative Design” OR “User-Centered Design” OR “Co-creation”

The search results were exported into Zotero (Corporation for Digital Scholarship) for reference management. Duplicate records were initially removed using Zotero’s automated deduplication feature, followed by a manual review by the first author (SBG) to identify and eliminate any remaining duplicates.

### Selecting the Studies

The inclusion and exclusion criteria were established before the study commenced and applied during the screening process (SBG, VP, and HP). Sources were included if they were (1) focused on developing or evaluating e-tools, and (2) discussed e-tool usability or user experience. Sources were excluded if they did not apply to the target population (older adults). To evaluate a larger range of studies, articles whose target group was 50 years or older were considered. Some studies define ’older adults’ as individuals aged 65 years and above, while others include individuals aged 50 years and older, especially in the context of research related to aging and technology use [25]. Considering individuals aged 50 years and older as “older adults” helped to retrieve a broader set of articles, particularly those focusing on promoting the use of e-tools for the aging population. This approach ensured that relevant studies addressing the needs of both younger and older segments of the aging population were included, capturing a more comprehensive view of user experience with transportation-planning e-tools. The first author (SBG) screened all identified sources by title and abstract, followed by a full-text review to assess their eligibility. To mitigate the risk of bias, 20% of the study selection process was independently reviewed and confirmed by co-authors (VP and HP).

### Charting the Data

A data charting form was developed by SBG and validated by VP, using a Microsoft Excel software grid that included source characteristics (authors, year of publication, title of the article, source of article, country, and context of the studies), the purpose of the studies (objectives and study orientation), and mapping of the methodology and evaluation of user experience (design approach, setting, type of data collection and analysis, type of usability evaluation, and sample size).

### Collecting, Summarizing, and Reporting Results

Both descriptive-analytical method and thematic analysis [26] were used to analyze all categories of data (characteristics of sources, purpose of the studies, and the reflection of the methodology and evaluation). Results were discussed, analyzed, and combined to identify the knowledge gap. Table 2 shows the list of selected articles.

**Table 2.** List of the selected articles.

Title	Year	Aim	Context	Design approach	Study setting	Type of methodology
A New Tourist Audio Guide Service for Elderly People Integrated in the Mobile Phone: Preliminary Results [27]	2010	Evaluating user experience	Tourism	—^a^	Laboratory	Qualitative and quantitative
A User-Centered Interface Design for a Pill Dispenser [28]	2020	Evaluating user experience	Health	User-centered design	Laboratory	Qualitative and quantitative
A User-centred Design Approach for Mobile-Government Systems for the Elderly [29]	2018	Evaluating user experience	Service provider	User-centered design	Laboratory	Qualitative and quantitative
Adoption and feasibility of a communication app to enhance social connectedness amongst frail institutionalized oldest old: an embedded case study [30]	2018	Evaluating user experience	Communication	Participatory design	Real-world environment	Qualitative and quantitative
B2C Websites’ Usability for Chinese Senior Citizens [31]	2014	Evaluating user experience	Shopping	User-centered design	Laboratory	Quantitative
Co-Creation with Older Adults to Improve User-Experience of a Smartphone Self-Test Application to Assess Balance Function [14]	2020	Developing or redesigning an e-tool	Health	Participatory design	Laboratory	Qualitative and quantitative
Creating a digital memory notebook application for individuals with mild cognitive impairment to support everyday functioning [25]	2019	Evaluating user experience	Daily planner	—	Laboratory	Qualitative and quantitative
Design and Development of an eHealth Service for Collaborative Self-Management among Older Adults with Chronic Diseases: A Theory-Driven User-Centered Approach [32]	2021	Developing or redesigning an e-tool	Health	ISO 9241‐210 standard for human-centered design	Real-world environment	Qualitative
Design and Usability Evaluation of Mobile Voice-Added Food Reporting for Elderly People: Randomized Controlled Trial [33]	2020	Evaluating user experience	Nutrition	User-centered design	Laboratory	Qualitative and quantitative
Design and development of a mobile app to support the care of the elderly [34]	2021	Evaluating user experience and developing or redesigning an e-tool	Health	User-centered design	Laboratory	Quantitative
Development and Field Testing of a Long-Term Care Decision Aid Website for Older Adults: Engaging Patients and Caregivers in User-Centered Design [35]	2020	Evaluating user experience and developing or redesigning an e-tool	Health	User-centered design co-design	Laboratory	Qualitative and quantitative
Evaluation of an App to Support Healthy Living by Older Adults [36]	2017	Developing or redesigning an e-tool	Nutrition	User-centered design	Laboratory	Qualitative and quantitative
Home Monitoring System for Comprehensive Geriatric Assessment in Patient’s Dwelling: System Design and UX Evaluation [37]	2021	Evaluating User Experience & Developing or redesigning an e-tool	Health	User-centered design, participatory design	Laboratory	Qualitative and quantitative
Human Factors Analysis, Design, and Evaluation of Engage, a Consumer Health IT Application for Geriatric Heart Failure Self-Care [38]	2017	Developing or redesigning an e-tool	Health	User-centered design	Laboratory	Qualitative and quantitative
Human-Centered Design Study: Enhancing the Usability of a Mobile Phone App in an Integrated Falls Risk Detection System for Use by Older Adult Users [39]	2017	Evaluating user experience and developing or redesigning an e-tool	Health	Human-centered design	Laboratory	Qualitative and quantitative
Improving Messenger Accessibility for Elderly Users using User Centered Design (UCD) Methods (Study Case: WhatsApp) [40]	2021	Evaluating user experience and developing or redesigning an e-tool	Communication	User-centered design	Laboratory	Qualitative and quantitative
Older Adults Can Successfully Monitor Symptoms Using an Inclusively Designed Mobile Application [41]	2020	Developing or redesigning an e-tool	Health	Inclusive design	Real-world environment	Qualitative and quantitative
Measure It Super Simple (MISS) activity tracker: (re)design of a user-friendly interface and evaluation of experiences in daily life [42]	2022	Evaluating user experience and developing or redesigning an e-tool	Health	User-centered design	Real-world environment	Qualitative and quantitative
Mobile App Prototype in Older Adults for Post fracture Acute Pain Management: User-Centered Design Approach [43]	2022	Evaluating user experience and developing or redesigning an e-tool	Health	Human-centered design	Laboratory	Qualitative
Participatory Design: Apps from The Older Adults to The Older Adults [44]	2021	Developing or redesigning an e-tool	Health	Participatory design	Laboratory	Qualitative
Promoting social and collaborative language learning among older adults in the digital era: Development and evaluation of a smartphone app prototype using a design-thinking approach [45]	2022	Evaluating user experience and eveloping or redesigning an e-tool	Learning	Specific type of user-centered design approach known as design thinking (DT)	Laboratory	Qualitative and quantitative
Requirements Elicitation to Develop Mobile Application for Elderly [46]	2017	Evaluating user experience	Not specified	User-centered design	Laboratory	Qualitative and quantitative
Sahayak: An Application for Social and Physical Well-Being for the Elderly [47]	2020	Evaluating user experience and developing or redesigning an e-tool	Social and physical well-being	User-centered design	Laboratory	Qualitative
Tailoring digital apps to support active ageing in a low income community [48]	2020	Developing or redesigning an e-tool	Physical activity	Co-design	Laboratory	Qualitative
The Developments and Iterations of a Mobile Technology-Based Fall Risk Health Application [49]	2022	Evaluating user experience and developing or redesigning an e-tool	Health	User-centered design	Real-world environment	Qualitative and quantitative
Untold Stories in User-Centered Design of Mobile Health: Practical Challenges and Strategies Learned From the Design and Evaluation of an App for Older Adults With Heart Failure [50]	2020	Evaluating user experience and developing or redesigning an e-tool	Health	User-centered design	Laboratory	Qualitative and quantitative
Usability and feasibility of consumer-facing technology to reduce unsafe medication use by older adults [51]	2020	Evaluating user experience	Health	User-centered design	Laboratory	Qualitative and quantitative
User Centered Design Approach for Elderly People in using Website [52]	2012	Developing or redesigning an e-tool	Transportation	User-centered design	Laboratory	Qualitative
User Interface for Social Networking Application for the Elderly [53]	2013	Evaluating user experience and developing or redesigning an e-tool	Social network	User-centered design	Real-world environment	Qualitative and quantitative
Usability testing of tablet-based cognitive behavioral intervention application to improve a simple walking activity for older adults with arthritis fatigue [54]	2021	Evaluating user experience and developing or redesigning an e-tool	Health	User-centered design	Real-world environment	Qualitative
The TV-WEB project—combining internet and television—lessons learnt from the user experience studies [55]	2017	Evaluating user experience	Entertainment	—	Laboratory	Qualitative and quantitative
Usability Study on Railway Self-Service Terminal Interface for the Elderly [56]	2018	Evaluating user experience	Transportation	—	Real-world environment	Qualitative and quantitative
Redesigning websites for older adults: a case study [57]	2014	Evaluating user experience and developing or redesigning an e-tool and documenting or extracting usability principles	Tourism	—	Laboratory	Qualitative and quantitative
Evaluating websites for older adults: adherence to ‘senior-friendly’ guidelines and end-user performance [58]	2008	Evaluating user experience	Not specified	—	Laboratory	Qualitative and quantitative
Evaluating 3D Printed VR Controller Prototypes to Increase VR Accessibility for Older Adults [59]	2022	Evaluating user experience	Not specified	—	Laboratory	Qualitative and quantitative
The Co-Design/Co-Development and Evaluation of an Online Frailty Check Application for Older Adults: Participatory Action Research with Older Adults [60]	2023	Evaluating user experience and developing or redesigning an e-tool	Health	—	Laboratory	Qualitative and quantitative
Applicability of the User Experience Methodology: Communication and Employment Web Portal for Older Adults [61]	2023	Evaluating user experience and developing or redesigning an e-tool	Communication and employment	—	Laboratory	Qualitative and quantitative
Integrated health system to assess and manage frailty in community dwelling: Co-design and usability evaluation [62]	2023	Evaluating user experience and developing or redesigning an e-tool	Health	Co-creation human-centered design	Real-world environment	Qualitative and quantitative
User-centered design (UCD) of time-critical weather alert application [63]	2023	Evaluating user experience and developing or redesigning an e-tool	Weather	User-centered design	Laboratory	Qualitative and quantitative
Web-Based Cognitive Behavioral Therapy for Depression Among Homebound Older Adults: Development and Usability Study [64]	2023	Evaluating user experience and developing or redesigning an e-tool	Health	Co-design	Laboratory	Qualitative and quantitative

a not available.

## Results

### Screening Results

**Figure 1.**
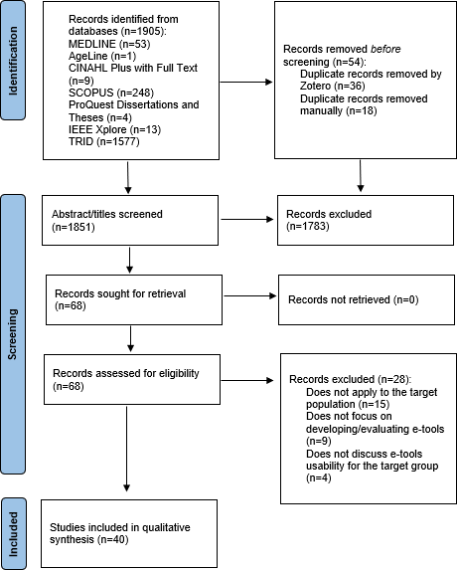
PRISMA (Preferred Reporting Items for Systematic Reviews and Meta-Analyses) 2020 flow diagram detailing the process of literature search, screening, and selection.

Overall, 1905 studies were identified through the databases. The record after duplicate elimination was 1854. In total, 68 sources were retained after screening for title and abstract. Finally, since 28 records did not meet the mentioned inclusion and exclusion criteria, 40 sources were included in this review (Figure 1; Checklist 1).

### Characteristics of Sources

Out of a total of 40 studies, 4 studies [27,52,57,58] were published during the first decade of the scope (2002‐2012) (10%), while 36 studies [14,25,28-51,53-56,59-64] were published after 2012 (90%). These studies were conducted across different continents, with 14 (35%) in Europe [14,27-29,32,36,37,39,42,45,53,55,57,62], 10 (25%) in Asia [31,33,38,40,44,46,47,52,56,60], 12 (30%) in North America [25,30,35,43,49-51,54,58,59,63,64], and 4 (10%) in South America [34,41,48,61]. The majority of the sources (29 studies) were peer-reviewed articles, representing 72.5% of the total [14,25,30,32,33,35-45,48-51,54,55,57,58,60-64], while the remaining 11 studies were conference articles (27.5%) [27-29,31,34,46,47,52,53,56,59].

Most contexts of the identified studies were related to health [14,28,32,34,35,37-39,41-44,49-51,54,60,62,64]. The other identified study contexts included tourism [27,57], shopping [31], daily planning [25], learning [45], social and physical well-being [47], physical activity [48], service provider [29], communication [30,40,61], nutrition [33,36], social network [53], entertainment [55], and weather [63]. In total, 3 articles did not specify the context of the study [46,58,59]. In total, 2 studies aimed to develop an e-tool and evaluate user experience in transportation [52,56].

### The Purpose of the Selected Studies

The purpose of the selected articles was categorized into three groups: (1) documenting or extracting usability design principles specified to older adults, (2) evaluating user experience, and (3) developing or redesigning an e-tool for older adults. Out of the 40 articles, only 1 (2.5%) followed all 3 objectives [57]. In total, 12 (30%) articles [25,27,29-31,33,46,51,55,56,58,59] focused on evaluating user experience, while 19 (47.5%) articles [28,34,35,37,39,40,42,43,45,47,49,50,53,54,60-64] concentrated on both evaluating user experience and developing or redesigning an e-tool. The remaining 8 (20%) articles [14,32,36,38,41,44,48,52] only focused on developing or redesigning an e-tool. Evaluating user experience had the highest rate among all the studies, representing 80% of the total (32 studies) [25,27-31,33-35,37,39,40,42,43,45-47,49-51,53-64].

### Empirical Methods of Usability Evaluation

#### Design Approach

Out of the total 40 articles, 22 (55%) of them were based on user-centered design [28,29,31-34,36,38-40,42,43,45-47,49-54,63], while 5 (12.5%) articles followed a co-design or participatory design approach [14,30,44,48,64]. In total, 3 (7.5%) articles used both user-centered design and co-design approaches [35,37,62], and 1 (2.5%) article followed an inclusive design approach [41]. There were 9 (22.5%) articles that did not specify any design approach [25,27,55-61].

#### Settings

Regarding experimental setting, 31 (77.5%) articles [14,25,27-29,31,33-40,43-48,50-52,55,57-61,63,64] were conducted in a laboratory setting, while the remaining 9 (22.5%) articles [30,32,41,42,49,53,54,56,62] took place in a real-world environment.

#### Type of Data Collection and Analysis

Most of the articles (31 studies) used both qualitative and quantitative methods for data collection and analysis [14,25,27-30,33,35-42,45,46,49-51,53,55-64], which represented 77.5% of studies. In total, 7 (17.5%) articles used the qualitative method [32,43,44,47,48,52,54], and just 2 (5%) articles [28,34] used only the quantitative method.

#### Usability Evaluation

Usability evaluation is usually divided into 2 general categories: (1) formative usability (usability inspection and expert-based): evaluating systems by experienced professionals using predetermined principles to identify usability issues [65], such as heuristic evaluation [66] and cognitive walkthrough [67]; and (2) summative usability (usability testing and user-based): evaluating the system by observing and recording the objective performance and subjective opinion of target users while interacting with a product to identify usability issues [68]. In usability tests, data are collected through diverse methods of objective performance and subjective opinions. A great number of studies (36/40) used summative usability evaluation (user-based), representing 90% of the studies [14,25,27-47,49-51,53,56-64]. Among the 40 articles, 10 articles (25%) used both summative and formative usability evaluation [29,39,44-46,50,57,58,61,62]. Only 1 (2.5%) article used formative usability evaluation [54], and 3 (7.5%) articles did not specify that they evaluated usability [48,52,55].

Data were collected through various methods during usability testing among older adults. Objective performance information was gathered from 19 articles, with task performance metrics being the most commonly used method [14,25,29,33,37,39,45,46,51,56-58,63], followed by observation 7 [14,30,40,42,43,47,64] and screen recording 3 [14,45,64] as less frequent methods. Subjective opinions (older adults’ experiences and their design preferences) were predominantly collected via questionnaires [14,25,27,29-31,33-39,41,42,45,50,51,53,57-60,62-64], with additional methods including think-aloud protocol [14,35,37,38,40,44,45,50,51,54], interviews [30,32,35,36,38,40,42,47,49-51,53,56,57], focus groups [27,28,59,60], and open-ended questions [33,36,37,43,58,64]. A subset of studies also used formative usability evaluation, some including heuristic evaluations [29,39,44-46,50,57,58,61], and cognitive walkthrough a few articles [54,62].

The questionnaire was the most frequently used data collection method. Among them, the System Usability Scale (SUS), measuring efficacy and satisfaction with usability when users perform specific tasks [69], was the most prevalent one. Table 3 demonstrates a structured summary of the usability evaluation methods and their frequency.

**Table 3.** Usability testing data collection methods.

Usability evaluation type	Data collection method	n (%)
Objective performance	Task performance metrics	13 (32.5)
	Observation	7 (17.5)
	Screen recording	3 (7.5)
Subjective opinions	Questionnaire System Usability Scale (SUS) (15 articles) After-Scenario Questionnaire (ASQ) (2 articles) Technology Acceptance Model (TAM) (3 articles) Post-Study System Usability Questionnaire (PSSUQ) (1 article) User Interface Satisfaction (QUIS) (2 articles) NASA Task Load Index (NASA-TLX) (2 articles) Health Information Technology Usability Evaluation Scale (Health-ITUES) (1 article) Self-Assessment Manikin (SAM) (1 article) Self-designed questionnaires (7 articles) Usability Metric for User Experience (UMUX) (1 article) Single Ease Question (SEQ) (1 article) User Experience Questionnaire (UEQ) (1 article)	26 (65)
	Think-aloud	10 (25)
	Interview Semi-structured (5 articles)	14 (35)
	Focus group	4 (10)
	Open-ended questions	6 (15)

### Sample Size

The number of participants varied, based on the purpose of the evaluation (ranging from 3 to 168). In the studies with a participatory or co-design approach (8 studies), the average sample size was 10, ranging from 3 to 22.

### Synthesis of Results

Most of the studies focused on developing eHealth, and only 2 articles [52,56] were related to transportation. The first one [52] was about the usability evaluation of an existing travel planning website via a user-centered design approach and did not concentrate on developing the website, while the other one [56] concentrated on evaluating the user experience of existing rail terminal interfaces for older people without using any design approach. The first one was conducted in a laboratory setting with 3 older adults via various qualitative methods, while the second one was conducted in a real-life setting with 5 older adults and 5 adults via a combination of both qualitative and quantitative methods. Although 1 of these 2 articles was performed in a real-life setting, this study aimed to evaluate older adults’ experience using existing services and did not concentrate on developing a transportation planning age-friendly e-tool.

## Discussion

### Principal Findings and Comparison With Previous Works

This scoping review aimed to identify knowledge gaps regarding the user experience design of older adults in using transportation planning e-tools. Our study reveals that e-tools designed for older adults have grown dramatically in recent years in health-related fields, while only 2 studies were related to transportation. Despite the large number of e-tools that have been developed to support transportation planning [70], documenting the user experience is rarely considered. This lack of focus may explain why it remains challenging for many older adults to use these e-tools effectively while planning their travels and finding their way from one place to another. For example, real-time travel information, as a crucial aspect of travel planning [71], is expected to significantly impact the user experience of older adults, by reducing the anxiety of getting lost [72], protect them from the weather traffic congestion [73,74] and enhance the use of modes of transportation that are tailored to their needs and preferences [75]. Statistics Canada [76] reported that only 54% of older adults have a smartphone for personal use, many of whom rarely use its various functions, which creates a gap between the intended goal of transportation planning e-tools and their current use. The lack of studies aiming to document older adults’ experience in using transportation planning e-tools might thus affect their accessibility in terms of content and functionality. In this context, Subasi et al [77] argue that developing a system that is not only universally accessible but that also meets the specific expectations of older adults will optimize their usage of the digital system.

This review also revealed the lack of studies concentrating on developing transportation planning e-tools using participatory design. This result might be explained by the challenges related to this design approach, such as substantial time, resources, and institutional commitment [78]. In participatory design, designers try to learn the realities of users’ situations while the users strive to articulate their desired aims and learn the appropriate technological means to acquire them [20]. Although it seems that participatory design is an approach to the user-centered design [79], it is actually a shift in attitude from designing “for” users (user-centered design) to designing “by” users (participatory design) [80]. To address the challenges of participatory design, it would be good to understand the optimal methods and timing for involving users in the study process. Providing concrete deliverables and regular updates [81] could motivate participants by demonstrating the tangible results of their contributions to the development of transportation planning e-tools. Additionally, establishing a welcoming and enjoyable atmosphere at the beginning of co-designed sessions, such as icebreaking [9], can help participants feel comfortable and build trust with the researchers. Simplifying some consultation sessions into questionnaires or virtual sessions could lessen the burden of in-person meetings for both older adults and the research team, particularly during winter. This strategy would also facilitate frequent and balanced engagement with older adults while reducing expenses [82].

Although the aim of a considerable number of studies was to evaluate the experience of older adults using existing or newly developed e-tools, only 1 [57] considered not only evaluating user experience and redesigning an e-tool but also documenting usability design principles. It should be noted that developing usability design principles and guidelines for age-friendly e-tools is crucial due to variations in older adults’ design characteristics [83]. Some user-centered methods, such as persona [84], might help to consider the needs and preferences of a wide range of older adults in developing age-friendly e-tools. More specifically, persona is used to identify user expectations through the definition of personality characteristics and specific personal details [84]. According to Cooper [85], the aim of persona is to have a comprehensive and global view of the target population to know for whom the product is not intended, and its approach is concentrating on “specific or canonical users” [84]. As none of the reviewed studies mentioned the use of persona, it would be interesting for future studies to explore how this method could help classify older adults with, for example, varying levels of digital literacy, diverse mobility profiles, and different travel planning habits. This could help identify variations in older adults’ design characteristics and aid in the development of usability design principles.

Based on our results, combining formative (expert-based) and summative (user-based) usability evaluation using a mixed-method and providing the intersectoral participation of different disciplines might help to perform a comprehensive evaluation of the transportation planning e-tools for older adults. Indeed, most of the sources reviewed (77.5%) used both qualitative and quantitative methods for data collection and analysis, which helps to gain a detailed understanding of the needs and preferences of older adults. As argued by Creswell and Plano Clark [86], using a mixed method could provide a deeper understanding of older adults’ expectations in travel planning via transportation planning e-tools that would help to further improve knowledge in the production of guidelines and—accordingly—develop useful e-tools. Furthermore, most of the articles conducted in standardized situations used summative usability evaluation (user-based testing), highlighting the urgent need to capture the experience of older adults, more specifically in real-life settings, to help design products as close to actual usage as possible [87]. However, conducting a summative usability evaluation in a real-life context is challenging, due to its resource-intensive and time-consuming nature, which makes it costly. To address these challenges effectively, it would be good to adopt a participatory design approach involving a minimum of 5 participants. These participants should encompass diverse mobility profiles, digital knowledge, and travel planning habits, representing the targeted end-users to address 80% of usability problems, as proposed by Nielsen [65].

### Limitations

This study identified knowledge gaps regarding the design user experience of older adults when using transportation planning e-tools. However, it has certain limitations. Although the data selection and extraction processes were confirmed by co-authors, study screening was performed by the first author. It is possible that some articles were not retrieved due to the selected keywords and lack of consideration for gray literature. Moreover, we did not use the methodology suggested by Levac et al [88], that includes a consultation step. This step would be practical in transferring development knowledge for e-tools for a broad spectrum of older adults through different disciplines such as gerontology, web developing, and design. Finally, although we did not systematically account for all plural forms, alternative spellings, or flexible word order, the use of controlled vocabulary (eg, MeSH terms) and keyword variations helped maximize the retrieval of relevant literature, while the librarian’s review ensured the rigor of the search strategy and adherence to best practices in systematic reviews.

### Conclusions

Supporting the mobility of older adults should be a priority in developing a transportation-related e-tool. According to this review, there are no studies aimed at specifically improving the mobility experience of older adults using an age-friendly transportation planning e-tool in a real-life context. Therefore, it is necessary to fill this gap by using a participatory approach (co-design) to better understand the needs of older adults in a real context to create age-friendly design principles and guidelines in the development of a transportation planning e-tool. This will not only improve their usability experience when planning a trip using a transportation planning e-tool but also strengthen their social participation.

## Supplementary material

10.2196/63273 Checklist 1 PRISMA-ScR Checklist.

## Abbreviations

PRISMA: Preferred Reporting Items for Systematic Reviews and Meta-Analyses
SUS: System Usability Scale
